# Review of Methodologies for Assessing Sustainable Diets and Potential for Development of Harmonised Indicators

**DOI:** 10.3390/ijerph16071184

**Published:** 2019-04-02

**Authors:** Paul Eze Eme, Jeroen Douwes, Nicholas Kim, Sunia Foliaki, Barbara Burlingame

**Affiliations:** 1 School of Health Sciences, College of Health Science, Massey University, Palmerston North 4442, New Zealand; n.kim@massey.ac.nz (N.K.); b.burlingame@massey.ac.nz (B.B.); 2 Centre of Public Health Research, Massey University, Wellington 6140, New Zealand; j.douwes@massey.ac.nz (J.D.); s.foliaki@massey.ac.nz (S.F.)

**Keywords:** sustainable diets, food systems, indicators, health, economic, nutrition

## Abstract

The underlying values and priorities that drive policy responses depend largely on the constructs that researchers and decision makers select to measure and the metrics used. Despite much recent attention being given to sustainable diets and food systems and to the importance of clearly measuring sustainability to meet targets, to achieve goals, and to appraise dietary and environmental policies, it is not commonly agreed how the different indicators of sustainable diets are assessed. The evidence base for assessment of these indicators are frequently weak, fragmented, and arbitrary. The aim of this paper was to compare a range of published methods and indicators for assessing sustainable diets and food systems in order to harmonise them. Keyword and reference searches were performed in PubMed, Scopus, CAB Abstracts, and Web of Knowledge. Fifty-two studies (21 proposed methods and 31 used methods) that combined environment, nutrition and health, and socioeconomic aspects of sustainable diets were reviewed. The majority (over 90%) of the studies focused on high-income countries. Twenty-eight studies assessed the environmental effects of different dietary practices, eight of the studies examined the nutrition and health indicators used for assessing sustainable food systems, and seven studies assessed the social and economic costs of diets. A classification of the elements was developed, and common elements are proposed for standardizing. These elements were categorized into nutrition and health indicators, environment indicators, and socioeconomic indicators. Standardized or harmonized indicators can be used for consistency and applicability purposes and to support, implement, and monitor relevant policies.

## 1. Introduction

The Food Agriculture Organization (FAO), World Health Organization (WHO), and United Nation University (UNU) [1] define diet as the set of food, beverages, and nutrients that are consumed by an individual or by a community of individuals during a certain period. A number of environmental, health-related, and socioeconomic factors can influence diets. The consideration of the interrelationships between these factors, particularly in the context of environmental resource limits, led to the concept of sustainable diets. According to FAO [2], sustainable diets are defined as those diets with low environmental impacts, which contribute to food and nutrition security and to a healthy life for present and future generations. Sustainable diets are protective and respectful of biodiversity and ecosystems, culturally acceptable, accessible, economically fair and affordable, nutritionally adequate, safe, and healthy while optimizing natural and human resources.

“All food systems are sustainable.” is the central policy objective of the UN’s Zero Hunger Challenge and an explicit feature of Sustainable Development Goal 2 (SDG 2) which seeks to “end hunger, achieve food security and improved nutrition, and promote sustainable agriculture.” Although they may appear straightforward, food systems and dietary patterns are in fact determined by a complex interplay of human, economic, social, environmental, and political factors. They can be difficult to define and characterise from any one perspective in terms of sustainability and may require multiple indicators for effective measurements.

In keeping with this, food systems have been defined and assessed in the literature from a range of perspectives [3,4]. At the same time, it is appreciated that a complete and encompassing definition should reflect the sum total of processes that link agricultural production to consumption, including food losses and waste, as well as the positive and negative impacts of these activities and processes on human and environmental health and wellbeing. The High Level Panel of Experts for the Committee on World Food Security states: A food system gathers all the elements (environment, people, inputs, processes, infrastructures, institutions, etc.); activities that relate to the production, processing, distribution, preparations, and consumption of food; and the outputs of these activities, including socioeconomic and environmental outcomes [5].

Globally, food security is threatened by the degradation of terrestrial, freshwater, and marine ecosystems and species used for medicine and food which are increasingly being neglected, underutilised, and ultimately lost. For example, according to FAO [6], approximately 200 million people are employed by the worlds’ fisheries, which contribute about 16 percent of the total protein eaten by the world. Nonetheless, approximately 80 percent of the world marine fish stocks, for which appraisal information is available, are overburdened, weakened, or recuperating from depletion. Bell and Taylor [7] reported that the Pacific Island Countries were facing many challenges to food security—accessibility, availability, and the utilization of nutritious foods. The authors outlined the causes of the challenges: fast population growth and urbanization; limited opportunities to acquire income; high levels of imported and processed foods high in salt, sugars, and fats; and the inability of communities to engage in small-scale agriculture production (SDG 2) due to a deficit of cultivable land. 

Diets as drivers of sustainable food systems have been discussed across many inter-sectoral bodies and interdisciplinary perspectives, resulting in mounting information and analytical research and a range of methodologies [8]. Despite being agreed upon, methodologies for quantifying sustainable diets show considerable variation with each other and may or may not be generally applicable, and resolving this variation is the rationale of the study. The harmonization of these indicators will contribute to the global monitoring of SDGs in addition to reporting on targets related to food systems and diets. This will ensure that the interconnected issues represented by different assessment targets are bridged as well as that the total amount of indicators needed for creating an extensive monitoring indicators framework for assessing sustainable food system is reduced. For example, the harmonization of gender indicators in Kyrgzstan strengthened the gender monitoring of the Millennium Development Goals (MDGs) in their different regions [9]. Some of the indicators have been field-tested more than others. In our view, there are two interrelated needs. Firstly, and to the extent possible, it would be desirable for the various methodologies to be harmonised. Secondly, methodological approaches for characterising sustainable diets should be field-tested in different regions to determine their specificity, appropriateness, and applicability. The need to characterise pressures that drive the divergence between current dietary patterns and sustainable diets is particularly urgent for populations with extreme and immediate vulnerabilities such as Small Island Developing States (SIDS). The main challenge in this area is that there are several diverse ways to aggregate different methods and indices for the purpose of harmonisation. In this paper, the aim of the study was to compare the different methodological frameworks proposed for assessing sustainable diets and food systems and to harmonize the proposed indicators.

## 2. Materials and Methods

**Figure 1:**
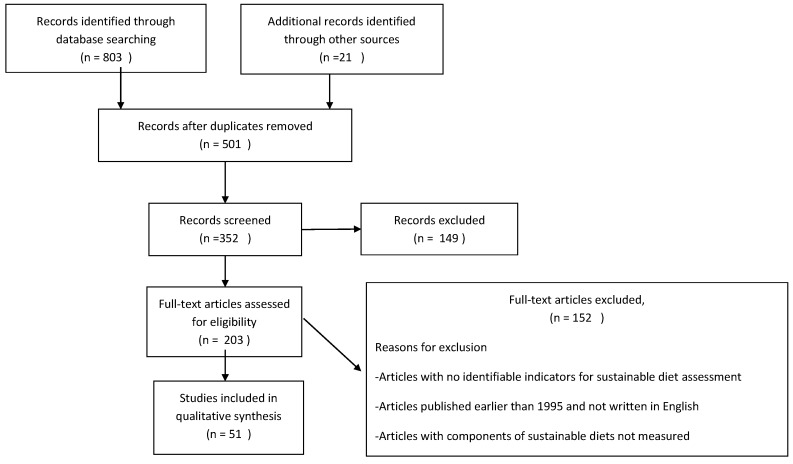
A flow chart of how the articles were selected and included in the review.

A literature search was performed in PubMed, Scopus, CAB Abstracts, and Web of Knowledge bibliographies between March 2017 and November 2018 using the search terms “sustainable food system” or “sustainable diet” and “assessment”, “health”, “environment”, “nutrition”, “social”, or “economics”. The search window was 1995–2018 with restriction to items published in English. Figure 1 shows the detailed process of the manuscripts included. Both peer-reviewed works and appropriate publications from the grey literature (such as conference proceedings and technical reports) were included, as long as they met all the following inclusion criteria: the quantification of environmental indicators linked to dietary intakes as greenhouse gas emissions, land use, or water use at a population level; the collection of dietary information to elucidate baseline diets at the national, household, or individual level; the estimation of the healthy aspects of sustainable diets; and the measurement of socioeconomic variables. Articles with no clearly identifiable indicators for assessing sustainable food systems, as well as review articles, were excluded. Potential papers meeting the inclusion criteria were accessed, and the details were extracted on the following variables: country of the study, main objective(s) of the paper, main findings, and main indicators identified. Only the full texts were included in the final analyses.

The harmonisation of the indicators was undertaken using the “interoperability cube” approach introduced by Mulder [10], specifically designed to explore and enable the harmonisation of methods and tools used in “living labs” research, including among the European Network of Living Labs (ENoLL). The cube builds on the idea that the main focus is on synergies and the parts that living labs wish to exchange with one another and with other forms for the harmonization of methods and tools. The cube identifies these exchange prospects and expressly defines these parts from structural, technical and discourse views. The more elements that match, the better the harmonisation. Up to five clusters can be used in the harmonization process. In this research, three of the clusters were used: user involvement, innovation outcomes, and methods and framework because they were deemed to be of the most relevance or applicability to the topic under investigation.

The “user involvement” indicators were designed according to an iterative approach [11]. The questions asked about the indicators included: “How to organize user involvement?”, “Who are the right users?” and “What is the efficacy level?” Users are important to define context-aware services, e.g., cultural differences. User context includes experience in the use of the indicator for data collection, the ability to apply and interpret the data using acceptable standard measures, and developing a strong interest in translating the set data into beneficial policies. Under the “innovation outcomes” cluster, the factors considered were the degree of flexibility of the indicators, user knowledge ability and the frequency of usage among the international agencies in measuring sustainable food systems. For “availability of the framework and methods”, the existence of reference standard methods and cultural preferences were considered. The indicators were classified under three categories: environment, nutrition and health, and socioeconomic.

## 3. Results and Discussion

This review covered 51 empirical studies of which only eight were published more than 10 years ago. The reviewed articles were classified under the categories of environment, nutrition and health, and socioeconomic. An inventory of the environmental indicators used for assessing sustainable diets across the different studies is provided in Table 1. A tabulation of the nutrition and health indicators is provided in Table 2, and the compiled socioeconomic indicators are shown in Table 3. A short list of harmonised indicators across these three categories is given in Table 4.

### 3.1. Potential for Geographic Bias

**Figure 2:**
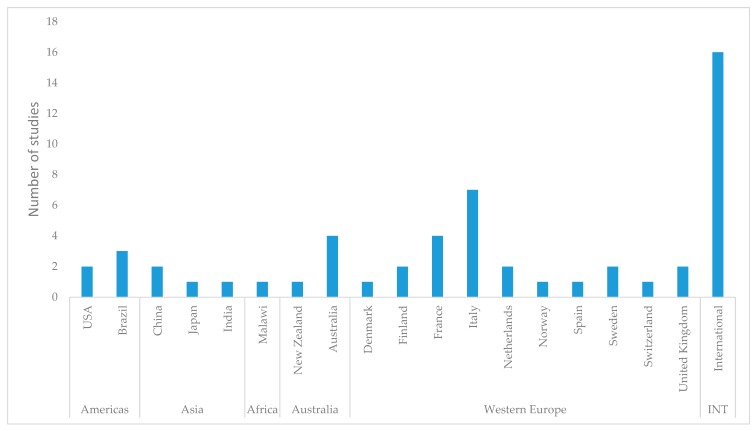
The distribution of the number of studies by countries and continents.

The majority (over 90%) of studies focused on high-income countries in western Europe and the USA (Figure 2). In spite of the fact that these high-income countries have substantially contributed to greenhouse gas emissions (GHGEs) related to food systems and agriculture, the detrimental effects of climate change and resource degradation shown in several of the measured segments of sustainability are likely to be felt most heavily in the low- and middle-income countries (LMICs) [12]. As evidence obtained from these high-income countries are being used by the governments of the LMICs to establish dietary guidelines, the problems and needs of these countries may not be effectively addressed.

### 3.2. Environmental Indicators

Thirty-two studies assessed the environmental effects of different dietary practices. These studies analysed the varying attributes of diets affecting land use, water, energy, planetary boundaries and many ecosystem services that were based on all the processes along food chain. Some of the environmental data in the studies showed decreased environmental footprints from the replacement of animal-based foods with plant-based foods, while others showed that plants had a higher footprint [13,14,15,16]. Whereas most studies showed lower environmental impacts from plant-based diets, a few studies showed a higher water footprint, and GHG emissions were observed from the replacement of calories from meat-reduction scenarios with increased plant-based foods [17,18]. Studies have shown that the formulation of substitute dietary patterns was also a factor in instances of higher environmental effects. For example, in Vieux et al. [19], meat reduction supplemented isocalorically by fruits and vegetables reflected a rise in emissions, while a secondary scenario of a replacement with mixed foods saw a net decrease.

In the use of an estimated Life Cycle Assessment (LCA) of diets, GHGE and the water footprint were the most common indicators measured (n = 26 studies; 41.1% of sample). This is consistent with previous reviews in related areas [20,21] which also identified LCA indicators as the most common assessment of dietary patterns. In their systematic review on the estimation of the potential to reduce GHGE and land use demands by varying the composition of the diet, up to 50% of the reviewed papers used GHGE as the indicator for measuring the environmental effect of diets. Environmental management system performance (n = 11 studies; 18.7% of sample) and land use, especially total per capita land requirement (n = 6 studies; 10% of the sample), were the second and third most frequently mentioned environmental indicators. Energy use, use of planetary boundary framework metrics and water use linked with the production and processing of foods were also commonly cited but in <5% of the studies.

### 3.3. Nutrition and Health Indicators

Twelve of the studies examined the nutrition and health indicators used for assessing sustainable food systems. Dietary intake assessments which include dietary diversity and dietary quality were the most common nutrition indicators assessed (n = 9 studies; 56.3% of sample). The Dietary Diversity Index, which is defined as the ratio of those obtaining a diverse diet to the overall population, is known as a promising indicator of dietary quality in the field of development economics [22] and, therefore, a relevant tool that could be used in other categories for measuring sustainable food systems. The other common indicators included the outcomes of focus group discussion, diet-related morbidity and mortality statistics, the rate consumption of local/regional foods, and the seasonality and rate of eco-friendly foods.

### 3.4. Socioeconomic and Other Indicators

The remaining eight studies assessed the socioeconomic indicators of diets at varying micro and macro levels. They focused on the value effects of purchasing power, the socioeconomic and lifestyle determinants, and the consumers’ preferences. The Price Index, income, wealth and equity indices were the most common socioeconomic indicators/indices used for measuring sustainable food systems. Most of these indicators identified could be used to measure the poverty index of a population. Research has shown that a sustainable diet is impacted not only by poverty but also by inequality [23]. Although, not part of the review because it is a trade–industry paper, the Sustainability Consortium has used a wide multi-stakeholder process to carry out a comprehensive “hot spot” analysis within the food supply chain and has identified potential societal indicators as affecters of sustainable food systems [24]. Other indicators include the agricultural production on sustainable food systems and the production area index.

### 3.5. Development of the Harmonised Indicators

Existing assessment indicators for measuring sustainable food systems in different categories are shown in Table 1, Table 2 and Table 3. These indicators were categorized by the general process concepts. The development of the harmonized model represents the overall maturation of reasoning behind the internationally recognized assessment models. The predominant idea is that the harmonized indicators were to be based on user involvement, innovation outcomes and the availability of reference standards. The structures of the harmonized indicators regarded as similar have been unified. For example, under environmental indicators, LCA was the main methodological tool for assessing the environmental impact of a product in its life cycle. Carbon footprint, water footprint and ecological footprint are indicators for measuring LCA in any food system and were all selected. Environment management performance indicators and land use were selected because of its frequent use in measuring the environmental impact of food system. Elements missing from one indicator but could be found in the other indicators have been added to the first one. For example, under the socioeconomic indicators in the harmonized tool, income, wealth and equity indicators can be used to obtain all the other indicators in the same category. Fruit and vegetable biodiversity and the nutrient/non nutrient composition of foods were new indicators added in the harmonized indicators because of the lack of published data in their usage in measuring sustainable food systems. The role of these two new indicators in measuring sustainable diets has not been extensively discussed. Azzini et al. [25] emphasized the importance of nutritional quality as an element in dealing with local food sustainability. Barre et al. [26] proposed the need for integrating nutrient bioavailability when identifying sustainable diets.

An assessment and comparison of the sustainability of food production and dietary patterns in different countries could be facilitated by the widespread use of either a single harmonised system as developed in this paper or a standardised set of core indicators.

A tool of this type may be used in two ways: either retrospectively for a review and intercomparison of existing studies or prospectively as the basis for new research. In both cases, the use of a harmonized framework offers a solution to the problem of bridging methods and tools from research undertaken in varying settings.

The field-testing of the framework is recommended to identify any significant omissions or weaknesses. Thus, the limitation of the study was that some of the harmonized indicators have not been field-tested and that the review was limited to only studies that identified indicators that were specifically proposed or used. Further research is proposed to enhance the specificity of certain indicators, as well as to expand the purview of that harmonization efforts to include other methodological approaches.

Although the harmonised framework is intended for a wide application, the development of this tool was intended to investigate sustainable food systems among specific Pacific Island countries and Small Island Developing States. At the time of publication, the method has been deployed in the island state of Kiribati. Also, although the use of these harmonized indicators provides an overall impression of progress, it is not practicable or meaningful to combine all diverse indicator measures into a single index.

## 4. Conclusions

The review of the indicators for assessing sustainable food systems and sustainable diet reflects how much work has been done in measuring the environmental, nutritional/health and socioeconomic aspects of sustainable diet. Most of the indicators identified have been applied especially in the developed nations. Therefore, in the context of operationalizing these different aspects when designing sustainable diets, it is important to recognize that the concept of sustainable diets is not limited to food and nutrition but that it is used across multiple fields, which includes environment, agriculture, animal sciences, social and economic sciences. These harmonized indicators are principally intended to communicate and highlight progress in measuring a sustainable food system, to identify specific priority areas where action is required and to inform multi-sectoral policy development to achieve many of the Sustainable Development Goals.

## Figures and Tables

**Table 1:** The environmental indicators used for assessing a sustainable diet.

Reference	Country	Objective of the Paper	Main Findings	Main Indicators/Index Identified
Wratten et al. [27]	New Zealand	Measuring of sustainability in agricultural systems	The “Selwyn Stewardship Monitoring Scheme” in New Zealand showed that the arable farm was the most efficient with meat and that farms that deals with dairy were considerably less efficient.	- Selwyn Stewardship Monitoring Scheme
Carlsson-Kanyama. [12]	Sweden	Determine the outcome of greenhouse gas emissions (GHGEs) on rice, dry pea, carrot, potato, tomato and pork production	Animal rearing and crop management practices were more relevant to environmental outcomes than other areas of the food supply chain.	- Life Cycle Assessment (LCA): Energy use
Jungbluth et al. [28]	Switzerland	Assess obstacles and choices for the purchase of foods that are environmentally friendly	The largest impact on lowering diet-related GHGEs was not buying air-transported products and meat consumption reduction.	- LCA
White [29]	International	Examines how the role of changes in diet across populations leads to inequality in the delivery of environmental impacts	An inequality in dietary energy distribution is linked with an inequality in the use of land to a lesser extent than meat-intensive diets.	- Ecological footprint - Gini coefficient - Depth of the food deficit - Dietary energy in the food supply - Per capita food supply variability
Gerbens-Leenes, Moll and SchootUiterkamp [30]	International	The use of environmental indicators for the production and sustainability of food systems	Three performance indicators were identified: energy, the total land and water requirement per kilogram of available food to be used by individuals, business sectors and companies.	- Depletion of resources - Quality of urban environment - Waste treatment - Environment management system performance
Moldan et al. [31]	International	To identify and describe composite indicators of environmental sustainability	A number of composite indicators were identified and described. which include the Environmental Sustainability Index (ESI), Dashboard of Sustainability (DS) and Wellbeing/Stress Index (WI)	- Environmental Sustainability Index - Dashboard of Sustainability - Wellbeing/Stress Index - Ecological footprint - Living Planet Index - Direct Material Consumption (DMC)
Gerbens-Leenes and Nonhebel [32]	International	Examine the association between agricultural land use and eating patterns	Eating patterns linked to greater wealth (i.e., cheese, fruits and meats) require more agricultural lands.	- Total per capita land requirements
Risku-Norja et al. [33]	Finland	Determine agricultural GHGE for 4 diet settings and organic production in comparison with industrial production	Organic production has a higher GHGE because of more cultivated acreage, and the main origin of GHGEs from agricultural production is the soil management practices.	- Per capita GHGE (production only)
Stehfest et al. [34]	International	Measure the effect of a dietary shift toward less meat on the environment	The emissions of methane and nitrous oxide would permit an increased carbon uptake, and consumption of less meat would productively scale down land use.	- Integrated assessment model framework
Smedman et al. [14]	Sweden	Evaluate GHGEs from producing different beverages	Milk has the highest GHGEs when compared to the GHGEs of other beverages.	- LCA
Carvalho et al. [15]	Brazil	Evaluate red- and processed-meat intake and the impact meat consumption has on diet attributes and the environment	Diet quality was inversely associated with meat intake in men. Meat consumption emitted greenhouse gas emissions of 18071988 tonnes of CO_2_ equivalents, which represent about 4% of the total CO_2_ emitted by agriculture.	- Brazilian Healthy Eating Index - 24 h dietary recall - Carbon footprint
Macdiarmid et al. [35]	United Kingdom	Determine the outcome of varied dietary options on GHGEs	The removal of meats and foods from dairy does not necessarily lead to a reduction of diet-related GHGE.	- LCA
Scarborough et al. [36]	United Kingdom	Models the effect of the three environmental scenarios on life loss from cardiovascular disease and cancer	The model showed that in Scenario 1 resulted in 36,910 deaths prevented per year, and Scenario 2 averted 1999 deaths per year, while Scenario 3 resulted in 9297 deaths delayed per year. A 19%, 9% and 3% reduction in GHGE characterised Scenarios 1, 2 and 3 respectively.	- LCA
Capone et al. [37]	Italy	Analysed the environmental cost of nonadherence to the Mediterranean dietary pattern from a water footprint perspective	A reduced total water abstraction is linked to an adherence to the Mediterranean dietary pattern.	- Water footprint
Liu and Zhang [38]	China	Proposing a methodological framework for measuring the sustainability level of main agricultural regions in China on regional and country levels	The balanced method yields lower sustainable values than the aggregate method and sensitivity analysis.	- Land quality index - Resource carrying Index - Ecological risk index - Intensity of land management
Masset et al. [39]	France	Identify the most frequently consumed sustainable diets by people daily	The diets were categorized into lower carbon diets, higher-quality diets and more-sustainable diets. Each of them had beneficial outputs, but the more-sustainable diets had the best outcome.	- LCA - PANDiet score - Diet cost - Energy density - Energy content
Masset et al. [40]	France	Identify foods using measures of sustainability dimensions	Foods such as meat and fish had the biggest negative impact on the environment. A low nutritional quality and a high price characterised food that had a high environmental impacts.	- LCA - Freshwater eutrophication - Score for the nutritional adequacy of individual foods (SAIN) - Score for disqualifying nutrients (LIM) - 2006 KantatWorldPanel French household consumer
Peano et al. [41]	Italy	To develop an indicator–based tool to monitor sustainability in agric-food systems using the Slow Food Presidia project approach	The Slow Food Presidia project increased all the dimensions of sustainability and, in particular, socioeconomic and cultural capital by preserving the environmental quality aspects of the food products.	- Ecosystem diversity - Species diversity - Genetic diversity - Water quality - Air quality - Erosion index
Van Dooren et al. [42]	International	Explore the relationship between nutritionally healthy and ecologically sustainable diets	Meat and dairy consumption were mostly responsible for low sustainability scores.	- LCA - Land use
EAT Initiative, Sustainable Development Solutions Network (SDSN)and Consultative Group for International Agricultural Research (CGIAR) [43]	International	Develop integrated indicators for Sustainable Food Systems and Healthy diets in the Post-2015 Development Agenda	Integrated indicators were developed in three thematic categories: sufficient, nutritional, varied and safe diets; climate-resilient and environmentally sustainable food production; and resilient and equitable food system.	- Per capita protein consumption and per capita land requirement for animal protein - Micronutrient deficiencies - Prevalence of moderate or severe food insecurity based on the food Insecurity Experience Scale (FIES) - Dietary Diversity Score - Carbon emissions from agricultural land use - Mean Species Abundance (MSA) - Consumptive greenhouse gas emissions from diets in tCO_2_eq per year - Area eutrophicated vs. total national area - Volume of blue freshwater consumed through diet per week - % of food loss and waste from food production to consumption and % of food waste recycled - Income of smallholder farmers and fishing communities
Gill et al. [44]	Brazil, China and India	Evaluate the environmental effects of dietary changes consistent with the nutrition shifts	Increases in cereal supply in China and India and beef production in Brazil increased GHGEs and had an effect on the phosphorus and nitrogen cycles, respectively.	- National availability indicators - Planetary boundaries framework metrics
Ruini et al. [17]	Italy	Present the Barilla centre for Food and Nutrition’s “Double Pyramid Model” in order to raise people’s awareness of the impact of the environment on food	A diet based on the principles of the Mediterranean Diet (MD), as suggested by Double Pyramid, generates a lower environmental impact compared to diets that are heavily based on daily meat consumption. Eating lower on the pyramid lowers the environmental impact.	- Carbon footprint - Water footprint - Ecological footprint
Aleksandrowicz et al. [45]	International	Review the evidence on changes on sustainable dietary pattern in relation to dietary intake on the environment variables	An animal-based restriction was directly related to a decrease in environmental footprints, and a dietary transition yielded a moderate gain in the all-cause mortality risk.	- LCA - Land use - Water use
Dernini et al. [8]	International	Assessment of sustainability of diets based on the MD	A standard set of information (definition, methodology, background, data sources, limitations of the indicators and references) was provided for thirteen nutrition indicators identified.	- Food biodiversity composition and consumption - Rate of local/regional foods and seasonality - Rate of eco-friendly food production and/or consumption - Adherence to the Mediterranean dietary pattern
Immacolata and Augusto [46]	Italy	Measured environmental sustainability in the food systems	The application of the method of LCA for the reduction of environmental shocks were related to the life of the product chosen (olive oil), and the decisions were related to interventions on processes, products and activities.	- Life Cycle Assessment (LCA)
Mertens et al. [47]	Netherlands	To categorize and summarize the different approaches to operationalise the health aspects of environmentally sustainable diets	Five approaches to operationalize the health aspects of the diet were identified: food item replacement; dietary guidelines; dietary quality scores; diet modelling techniques; and a diet-related health impact analysis.	- LCA - Eco-indicator - Total ecological footprint - Land use - Energy efficiency - Water footprint
Pires et al. [48]	International	Evaluate how indicators related to water use and management perform against a set of sustainability criteria	Twenty-four indicators comply with the majority of the sustainability criteria; 59 indicators comply with two sustainability criteria, while 86 indicators fulfill just one of the four sustainability criteria; and one indicator does not fulfil any of the sustainability criteria.	- Water footprint - Access to safe drinking water - Existence of legislation advocating for Dublin principles of water - Fresh water living planet index
Pellicer-Martinez and Martinez [49]	Spain	The use of a water footprint (WP) to assess environmental sustainability in water resources at the river basin level	“Blue water” use is not sustainable due to a generalized overexploitation of aquifers, and surface water pollution is mainly caused by phosphate concentration.	- Water footprint
Seconda et al. [50]	France	Draw up a comparative description of four diets differing in the level of organic food consumption and the adherence to the Mediterranean Diet (MD) using multidisciplinary indicators to assess the sustainability of these diets	The adherence to nutritional recommendations was highest among the organic consumers and Mediterranean diet followers, lower among conventional consumers and Mediterranean diet followers and the lowest among conventional consumers and non-Mediterranean diet followers.	- Diet quality Index based on the Probability of Adequate Nutrient Intake PANDiet - Dietary Diversity Score - mPNNS-GS - Literature-based adherence score of Mediterranean diet
Dooren et al. [51]	Global	Identify a set of important indicators to assess the most pressing environmental impacts of diets	At the global and national levels, the planetary boundaries and the footprint approaches were respectively used to identify indicators, while the LCA was used at the product (micro) level.	- Climate impact - GHGE - Land use - Energy footprint - Water footprint - Carbon footprint - Ecological footprint
Kramer et al. [18]	Netherlands	Measure the performance of food products in a sustainable diet based on the balance of their contribution to nutrient intake and environmental impact, within the context of the Dutch diet	Increasing amounts of dairy in the optimized diet were associated with a steep increase in the environmental impact and in meat. Bread and breakfast cereals are sources of nutrients with a better environmental performance compared to dairy or meat within the context of the Dutch diet.	- Carbon footprint - Nutrient balance metrics - GHGE - Fossil energy use - Land occupation
Barre et al. [26]	France	Assess the impact of nutrient bioavailability and coproduction link considerations on dietary changes needed to promote a sustainable diet with a special focus on meat	The “fruits and vegetables” and “starches” quantities increased in all the modelled diets compared to the mean observed French diet.	- Bioavailability estimation - Nutrient calculation using food composition databases - GHGE - Atmospheric acidification - Marine eutrophication - Diet cost analysis
Osita et al. [52]	Japan	Examined the impact of changes in a Japanese diet from 1961 to 2011 and the effect of alternative diets on the nitrogen footprints of food	The 1975 Japanese diet, a balanced omnivorous diet, was reported to delay aging, with a protein content similar to the current level, and to reduce the current food nitrogen footprint (15.2kg N) to 12.6 kg N, which is comparable to the level in the protein diet (12.3 kg N).	- Nitrogen footprint

**Table 2:** The nutrition and health indicators used for assessing a sustainable diet.

Reference		Objective of the Paper	Main Findings	Main Indicators/Index Identified
Schacht et al. [53]	Norway	Determine consumers’ preference of fish with different origins and management practices	Farmed and wild salmon were least accepted while fish fed with a feed of plant origin were more accepted compared to other fishes.	- Sensory Evaluation Index
Pearson [54]	Australia	Determine the consumers’ dietary preferences in choosing organic foods	Greater than half (54%) of the respondents expressed readiness to increase the organic consumption, and 3% of them reported a high anticipation in the purchases of organic foods.	- Analysis of online questionnaire of self-selected adult food shoppers
WHO [55]	International	Measured health indicators of sustainable agriculture, food and nutrition security	The health indicators identified and linked to nutritional status, food quality and trade policies and programmes.	- Health outcome indicators such as prevalence rates - Food access and dietary quality indicators which include Household Dietary Diversity and the prevalence/incidence of food borne disease outbreaks - Food market/trade policies indicators
Dixon and Isaacs [56]	Australia	Assess consumer views on sustainable and healthy diets	Food purchase decisions were mainly influenced by cost, availability and family responsibility and not necessarily by sustainability or healthy foods.	- Focus group results - Ethnography results
Luckett et al. [57]	Malawi	To estimate and examine the role of household production and market acquisitions in providing dietary diversity to farm households in Malawi	Households further from roads and population centres had lower diversification (*p* < 0.01) and spread through comparatively more of their diversity from household production than households closer to market centres (*p* < 0.01).	- Nutritional Functional Diversity Score
Harry et al. [58]	Australia	Assess the dietary assessment method of sustainable dietary behaviour using a mobile food record (mFR) application	The use of mFR images for assessing fruit and vegetables, eggs, red meat and poultry was developed and tested for validity and reliability.	- mobile food record (mFR)
Benedetti et al. [59]	Italy	Assess the current dietary patterns among Italians, and analyse the effect of socioeconomic and lifestyle factors on Mediterranean diet constancy	Of all the socioeconomic characteristics, education proved to have a central role in determining the adherence to MD. Individuals with at least 8 years of education increase from the lowest (39%) to the highest (44%) category of the Mediterranean score.	- Food frequency questionnaire approach - Mediterranean Composite score
Dernini et al. [60]	International	Assessment of the sustainability of diets based on the MD	A standard set of information (definition, methodology, background, data sources, limitations of the indicators and references) was provided for thirteen nutrition indicators identified.	- Vegetable/animal protein consumption ratios - Average dietary energy adequacy - Dietary Energy Density Score - Nutrient density of diet - Food quality - Fruit and vegetable consumption/intakes - Dietary Diversity Score - Diet-related morbidity/mortality statistics - Nutritional anthropometry - Physical activity/physical inactivity prevalence
Benedetti et al. [61]	Italy	Determine the current food patterns of Italians using a composite indicator, and establish which of the indicators had a higher adherence to Mediterranean diet in Italy	Education, the tendency to practice sports on a regular basis and the ability to have breakfast and lunch at home positively impact people’s adherence to the Mediterranean diet.	- Mediterranean Diet Index: frequencies of consumption of 14 types of food (12 food groups plus 2 types of oils and/or fats)
Springmann et al. [16]	Global	Examined three different approaches to sustainable diets.	Animal-source replacement with plant-based ones were efficient, especially in improving nutrient levels, decreasing untimely mortality and lowering the environmental impacts.	- Nutrient content calculation - Replacement of 25–100% animal source foods with plant-based ones at a constant total calorie intake
Lachat et al. [62]	Global	Assessed the relationship between food biodiversity and diet quality of women and young children using diet species richness for wet and dry seasons	The dietary species richness showed stronger and more consistent associations with the diet quality indicators (Mean Adequacy Ratios and Dietary Diversity Scores) than Simpson’s index of Diversity index and Functional Diversity.	- Simpson’s index of diversity (represents number of different species consumed)
				- Functional diversity - Nutrient adequacy ratios
				- Mean adequacy ratios - Dietary diversity Score - Minimum Dietary Diversity
Vieux et al. [19]	Europe	Determine if the dietary changes needed to improve diet sustainability are similar across some European countries	Nutritional adequacy was not necessarily associated with a reduced GHGE, and maximum GHGE reductions attainable were filed from 62 to 78% with a minimal weight change of 2.8 Kg/day from the observed diet.	- GHGE - Diet weight - Energy weight - Mean absolute quantity variation of food items

**Table 3:** The socioeconomic indicators used for assessing a sustainable diet.

Reference		Objective of the Paper	Main Findings	Main Indicators/Index Identified
FAO (Food Agriculture Organization) [63]	International	Assess sustainability in the Food and agriculture sector	The sustainability monitoring and assessment routine (SMART) was developed to be used by companies and the agriculture sector.	- Investment Index - Vulnerability Index - Product Quality and Information Index - Local Economy Index - Cultural diversity
Jensen and Poulsen [64]	Denmark	Assess the economic effects for the New Nordic diet consumer compared with an average Danish Diet	The New Nordic Diet was about 17% more expensive than the Average Danish Diet when the energy content of the diet was adjusted and 25% more costly when there was no adjustment.	- Cost Index
Lombardini and Lankoski [65]	Finland	Assess the consequences of forced food choice restriction in schools on students’ diet	The effects were manifested in a decrease in the number of people who took part in school lunches and in the quantity of food taken to the plate and in an increase in plate waste.	- Food record - Lunch participation rate
Peano et al. [41]	Italy	Develop an indicator-based tool to monitor sustainability in agric-food systems using the Slow Food Presidia project approach	The Slow Food Presidia project increased all dimensions of sustainability and, in particular, socioeconomic and cultural capital by preserving the environmental quality aspects of the food products.	- Supply chain - Price - Production Area Index
Barosh et al. [66]	Australia	Assess the affordability of a typical compared to a healthy and sustainable food basket in Greater Western Sydney, Australia	Healthy and sustainable food basket was more costly than the typical basket in all five socioeconomic neighbourhoods studied.	- Price Index (price per unit weight of food items)
IOM (Institute of Medicine) and NRC (National Research Council) [67]	USA	Assess the social and economic effects of the U.S. system	Major classes of social and economic effects that can be linked to characteristics of the U.S. food system were outlined.	- Income, Wealth and Equity Indices - Quality of life indicators - Food costs and expenditures indicators - Food security indices - Food quality indices
Gustafson et al. [68]	USA	Develop a methodology on the concept of sustainable nutrition security using different metrics	Seven metrics for characterizing sustainable nutrition outcomes of food systems were proposed and developed using multiple indicators.	- Gender equity - Extent of child labour - Respect for community rights - Animal health and welfare
Barone et al. [69]	Brazil	Investigating the association between sustainability and foods, and to identify consumer’s perspective about the characteristics of sustainable and unsustainable foods	The terms “healthy diet” and “sustainable production” stood out in the sustainable diets concept. A higher educational level of the participants linked food to the natural environment and sustainability while individuals with lower educational levels associated food with source, nutrition and health.	- Questionnaire with word association, free listing and sentence completion tasks

**Table 4:** The harmonized indicators for assessing sustainable food system.

Nutrition and Health Indicators	Environment Indicators	Socio-Economic Indicators
Diet-related morbidity/mortality Dietary Diversity/Nutrient adequacy ratios Nutritional anthropometry/body composition Physical activity/inactivity prevalence Nutrients and Non-nutrient assessment of some commonly consumed foods	Ecological footprint Carbon footprint Water footprint Rate of local/regional foods and seasonality Environmental management system performance Fruits and vegetables biodiversity Land use	Income, wealth and equity indicators
